# Gestural acquisition in great apes: the Social Negotiation Hypothesis

**DOI:** 10.1007/s10071-017-1159-6

**Published:** 2018-01-24

**Authors:** Simone Pika, Marlen Fröhlich

**Affiliations:** 1 Department of Primatology, ‘Virtual Geesehouse’, Max Planck Institute for Evolutionary Anthropology, Deutscher Platz 6, 04103 Leipzig, Germany; 2 Anthropological Institute and Museum, University of Zürich, Winterthurerstrasse 190, 8057 Zurich, Switzerland

**Keywords:** Communication, Gestures, Acquisition, Social Negotiation Hypothesis, Chimpanzees

## Abstract

Scientific interest in the acquisition of gestural signalling dates back to the heroic figure of Charles Darwin. More than a hundred years later, we still know relatively little about the underlying evolutionary and developmental pathways involved. Here, we shed new light on this topic by providing the first systematic, quantitative comparison of gestural development in two different chimpanzee (*Pan troglodytes verus and Pan troglodytes schweinfurthii*) subspecies and communities living in their natural environments. We conclude that the three most predominant perspectives on gestural acquisition—Phylogenetic Ritualization, Social Transmission via Imitation, and Ontogenetic Ritualization—do not satisfactorily explain our current findings on gestural interactions in chimpanzees in the wild. In contrast, we argue that the role of interactional experience and social exposure on gestural acquisition and communicative development has been strongly underestimated. We introduce the revised Social Negotiation Hypothesis and conclude with a brief set of empirical desiderata for instigating more research into this intriguing research domain.

## Introduction

Language, more than anything else, distinguishes us from the rest of the animal kingdom (Knight et al. 2000). It provides us with the ability to express anything we think and to communicate these thoughts via a set of mutually comprehensible signals (Fitch 2010). Although the core features of human language are still highly debated (e.g. Jackendoff 2002; Pinker and Bloom 1990), one influential breakdown was provided by Hockett in the middle of the last century (Hockett 1960, 1963). Hockett thought of language as a collection of ‘design features’ suited to different tasks. He isolated nine (plus three additions made later) shared with other animals (e.g. interchangeability, semanticity, arbitrariness) and four features unique to human language:

1. Displacement, which refers to the ability to encode and understand meanings about past, future, or distant referents.
2. Productivity/openness, which denotes the skill to invent and understand new utterances/signal combinations.
3. Duality of patterning, which refers to the ability to combine meaningless units (phonemes) into meaningful ones (morphemes), which can then be combined into larger meaningful units (e.g. sentences).
4. Traditional (cultural) transmission, which denotes the fact that languages (or communication systems) are learned and not genetically encoded (Fitch 2010; Hockett 1960, 1963).

Specifically, the last feature—traditional transmission—was highly influential in the field of animal communication and resulted in a considerable amount of research on vocal learning (Catchpole and Slater 1995; Janik and Slater 1997; Snowdon 2017). This research bias into the vocal modality was also due to the fact that Hockett wrote before it was widely acknowledged that signed language is comparable to spoken language and that language forms an integrated system out of speech and gesture (McNeill 1985).

Interest in the learning processes underlying gestural signalling, however, dates back to the figure of Charles Darwin, who wrote the first scientific treatise on the subject—*The expression of the emotions in man and animals* (Darwin 1872). Besides providing a wealth of observations of human and nonhuman animal expression he, for instance, noted:

Englishmen are much less demonstrative than the men of most other European nations, and they shrug their shoulders far less frequently and energetically than Frenchman or Italians do. The gesture varies in all degrees from the complex movement, just described, to only a momentary and scarcely perceptible raising of both shoulders. […] I have never seen very young English children shrug their shoulders, but the following case was observed with care by a medical professor and excellent observer, and has been communicated to me by him. The father of this gentleman was a Parisian, and his mother a scotch lady. His wife is of British extraction on both sides, and my informant does not believe that she ever shrugged her shoulders in her life. His children have been reared in England, and the nursemaid is a thorough Englishwoman, who has never been seen to shrug her shoulders. Now, his eldest daughter was observed to shrug her shoulders at the age of between sixteen and eighteen months; mother exclaiming at the time, ‘Look at the little French girl shrugging her shoulders!’ […] The habit gradually wore away, and now, when she is a little over four years old, she is never seen to act thus. […] This gentleman’s second daughter also shrugged her shoulders before the age of eighteen months, and afterwards discontinued the habit. […] In this latter case we have good instance […] of the inheritance of a […] gesture; for no one, I presume, will attribute to mere coincidence so peculiar a habit as this, which was common to the grandfather and his two grandchildren who had never seen him. (Darwin 1872, pp 266–267)

This quote, although interesting from several perspectives (for instance gestural variability and cross-cultural differences; Eibl-Eibesfeldt 1968; Iverson et al. 2008; Morris 1979; Pika et al. 2009), suggests that the production of some gestures are innate, with gesture continuity and usage being highly influenced and shaped by the social environment.

Surprisingly, more than a hundred years later, we still know relatively little about gestural acquisition and underlying learning processes across the animal kingdom. Some suggest that humans’ earliest gesture types (ritualizations, deictic gestures, conventional gestures; see Box 1 for definitions) (Bates et al. 1975; Nicoladis et al. 1999; Petitto 1988) involve different learning mechanisms (Tomasello 1999). Others argue that human gestures are learned primarily through interactive routines with experienced conspecifics (Acredolo and Goodwyn 1988), or—with the exception of conventional gestures—represent spontaneously produced highly flexible communicative means (Child et al. 2014; McNeill 1992). Comparative research into gestural acquisition has so far been mainly focusing on natural communicative interactions of our closest living relatives, the great apes, and has resulted in a lively debate (Genty et al. 2009; Halina et al. 2013; Hobaiter and Byrne 2011b; Liebal and Call 2012; Perlman et al. 2012; Pika 2014).

**Box 1:** Glossary

*Audible gestures* Gestures that generate sound while being performed and also involve a visual component (Pika et al. 2003; Tomasello et al. 1994). *Bimodal signal* The combined production of two signal modalities, e.g. a gesture and a vocalization (Luef and Pika 2017). *Conventional gestures* (also called emblems, Ekman and Friesen 1969; or quotable gestures by others, Kendon 1992) Gestures, whose form and meaning are established by the conventions of specific communities and/or groups (e.g. waving goodbye) (Bates et al. 1979; McNeill 1992).
*Group/population*-*specific gestures* Gestures that are produced by the majority of individuals in one group/population, but are absent in another group/population (Goodall 1986; McGrew and Tutin 1978; Pika et al. 2003).
*Iconic gestures* Gestures that are related to their referent by virtue of some actual physical resemblance between the two (e.g. drawing a circle in the air to depict the shape of the sun) and are usually not interpretable without the accompanying speech (Feyereisen and de Lannoy 1991; McNeill 1992).
*Idiosyncratic gestures* Gestures that are produced by single individuals of the group/community only (Tanner and Byrne 1996; Tomasello et al. 1994).
*Intentionality* To qualify as first-order intentional behaviour, signallers have to act in a goal-directed way, produce voluntary, recipient-directed signals as a means to reach the desired goal, with the signalling behaviour eliciting a change in the recipient’s behaviour (Bruner 1981; Dennett 1983; Leavens and Hopkins 1998; Russell et al. 1997).
*Matrisyncratic gestures* Gestures observed in individuals of a single maternal family line only (Hobaiter and Byrne 2011b).
*Meaning of gestures* The meaning is identified as the response selected by the recipient from all of the responses open to it, which satisfies the signaller (e.g. the signaller stops signalling) (Cartmill and Byrne 2010; Hurford 2007; Lancaster 1975; Smith 1965).
*Object*-*associated gestures* Gestures that embody the use of mobile and immobile objects (Bates et al. 1989; Fröhlich et al. 2016a). *One*-*way gestures* Gestures that are produced by a single individual of a given dyad only (Tomasello et al. 1994).
*Ritualization* The signaller uses an effective behaviour for a request (e.g. children throwing their arms in the air to be picked up; Clark 1978; Lock 1978).
*Self*-*handicapping gestures* Gestures that involve signalling postures (e.g. lying in a supine position) that reduce the signaller’s probability of achieving its tactical objective in play (Fröhlich et al. 2016a; Hayaki 1985; Spinka et al. 2001). *Social learning* Indicates situations in which one individual attempts to actually reproduce or match the behaviour of another (Bandura 1986; Carpenter and Call 2002). *Symbolic gestures* Gestures that comprise whole-body enactments to depict actions and objects. They are associated with a referent metonymically (in that the gesture refers to an element or attribute of something, e.g. putting a finger to the nose and raising it for ‘elephant’) or are based on their mutual iconic relation to each other (e.g. flapping one’s arms to represent a bird’s wings; Acredolo and Goodwyn 1988; Namy and Waxman 1998; Pizzuto and Volterra 2000).
*Tactile gestures* Gestures that involve physical contact with the recipient (Pika et al. 2003; Tomasello et al. 1994).
*Visual gestures* Gestures that generate a mainly visual component without any physical contact or consistent audible component (Pika et al. 2003; Tomasello et al. 1994).

The aim of the present paper is threefold: first, we will briefly summarize the three most predominant perspectives on gestural acquisition in nonhuman animal communication—Phylogenetic Ritualization, Social Transmission via Imitation, and Ontogenetic Ritualization (see Table 1)—and the related findings and criticisms. Second, we will introduce the revised Social Negotiation Hypothesis (sensu Plooij 1978; Wittgenstein 1953) and discuss it in the light of our recent findings on gestural signalling in two chimpanzee communities and subspecies in the wild. The revised hypothesis (Fröhlich et al. 2016b) postulates that the creation of gestures does not begin with shortening of a functional action sequence (contra the Ontogenetic Ritualization Hypothesis), but the shaping (contra the Phylogenetic Ritualization Hypothesis) and exchange of full-blown behaviours. This exchange results in a shared understanding that certain behaviours

**Table 1:** Predictions for gestural repertoires and production based on the three predominant perspectives on gestural acquisition

	Repertoire concordance within groups	Repertoire concordance between groups	Idiosyncratic gestures	Group-specific gestures
Phylogenetic Ritualization	High	High	Absent	Absent
Social Transmission	High	Low	Absent	Present
Ontogenetic Ritualization	Low	Low	Present	Absent

- can be used communicatively;
- carry distinct meaning linked to particular social contexts; and
- are produced to achieve distinct goals.

The accumulated knowledge can be generalized across dyads (contra the Ontogenetic Ritualization Hypothesis), to enable the most efficient and least costly communication transfer, and is open to subsequent adaptation (e.g. a gesture type might first be used to initiate play but later to impress a possible rival; Pika 2014). Thus, each gestural performance by a given signaller represents a highly variable online adjustment (sensu Perlman et al. 2012). Third, we will present a brief set of empirical desiderata for instigating more research into this crucial research domain and spur more studies in other species (and potentially falsify our claims).

## Traditional routes of gestural acquisition

### The Phylogenetic Ritualization Hypothesis

The Phylogenetic Ritualization Hypothesis postulates that signals evolved from functional action sequences that previously had no communicative function (Darwin 1872; Tinbergen 1952). They are seen as *derived activities,* ‘borrowed’ from other contexts, which underwent some modification to accomplish a communicative and thus new function. Based on this ethological concept, the snarl of a wolf—a facial expression combined with a vocalization—derived from the practical action of retracting the lips to bite. A conspecific interacting with an ancestral wolf might have gained an advantage from reading this intention movement and anticipating the ensuing bite. In turn, a later generation of wolves using this lip retraction could benefit by triggering other animals to respond as if to an oncoming bite, and so, over many generations lip retraction may have evolved into the ritualized snarl observed in contemporary wolf communities. Although traditionally believed to play a major role in the evolution of various facial expressions, displays, and vocalizations (Van Hooff 1972, 2012), Byrne et al. (2017) argue that phylogenetic ritualization is also fundamental to the gestural signalling of great apes. This view predicts that gestural *production* is constant in form throughout development, with the repertoire sizes of species being highly uniform, although flexible in their *usage*. Evidence, supporting the importance of phylogenetic ritualization for gestural output, stems from studies of the ‘St. Andrews group’ using quantitative data of four groups of gorillas (*Gorilla gorilla*; three captive, one wild group; Genty et al. 2009), one wild chimpanzee (*Pan troglodytes*) community (Hobaiter and Byrne 2011b), and two adjacent wild bonobo (*Pan paniscus*) groups (Graham et al. 2017). The researchers suggested that gestural repertoires of great ape species are not only species typical but also ‘family typical’, with overlap of around 24 gesture types recorded in all genera. The most recent study even showed an overlap of 80% between gestural repertoires of two bonobo groups (the *E1* and the *P* community at *Wamba*, Luo Scientific Reserve, DRC) and a chimpanzee community (the *Sonso* community, Budongo Conservation Field Station, Uganda; Graham et al. 2017). The observed differences in gestural expressions concerned two distinct communicative functions—to solicit sex (higher number of gesture types in bonobos) and to display dominance/status (higher number of gestural types in chimpanzees). In addition, a study on gorillas (Redshaw and Locke 1976) and chimpanzees (Berdecio and Nash 1981) in captivity showed that individuals that had never seen another conspecific performing a signal, or could only interact with same-aged peers, produced gestures such as chest beat^1^ and slap. Studies on individuals living in groups with a more natural composition, however, revealed that the function and usage of these two gesture types were more diverse (Pika et al. 2003; Tomasello et al. 1994). These findings indicated a crucial influence of the social environment and development on the appropriate use of these signals. Similarly, studies on gestural production in several captive groups of all four great ape species showed that the degree of concordance in gestural repertoires (all gestures observed during the time of the respective data collection period) were relatively similar within and between groups. While gorillas displayed relatively high levels of gestural concordance across individuals and groups (Pika et al. 2003), bonobos, chimpanzees and orangutans (*Pongo pygmaeus*) had relatively low levels of concordance between individuals (Liebal et al. 2006; Pika et al. 2005; Tomasello et al. 1994).

### Social Transmission via Imitation

An alternative route to gesture acquisition is that individuals may acquire their gestural signals during their lifetimes rather than inheriting a biologically ‘hard-wired’ gestural repertoire as postulated above. Historically, social learning (see Box 1) via imitation has been considered an important route of gestural acquisition in great ape signalling (Liebal and Call 2012; Tomasello et al. 1994). In this process, gestures and their meanings are learned by individuals living in the same group that (a) understand the communicative intention of a gesturing individual, and (b) subsequently engage in role reversal imitation to produce the gesture type themselves when they have ‘the same’ communicative intention (Pika 2008; Tomasello 1999). This hypothesis predicts high degrees of gestural uniformity among individuals and within groups, paired with substantial differences between groups [e.g. group-specific gestures (see Box 1) or different usage; Call and Tomasello 2007]. Potential candidates for socially transmitted gestures are behaviours such as leaf clipping (Nishida 1980), and the grooming hand clasp (McGrew and Tutin 1978) reported in chimpanzee communities living in their natural environments and in some captive groups and a sanctuary (grooming hand clasp: van Leeuwen et al. 2012; Bonnie and de Waal 2006). These gesture types have been described to be absent at some sites and/or groups and to differ in their form and function (Ghiglieri 1984; McGrew and Tutin 1978; Nishida 1987; Sugiyama 1981). For instance, the grooming hand clasp has only been observed as being customary at the long-term study sites *Kanyawara*, *Mahale*, *Ngogo*, and *Taï* but not at *Bossou*, *Budongo*, and *Gombe* (McGrew and Tutin 1978; Whiten et al. 1999; *Ngogo*: S.P. personal observation). leaf clipping consists in some communities of ripping parts of the leaf with the mouth (Nishida 1980; Sugiyama 1981), while in other communities individuals use both mouth and fingers (Luncz and Boesch 2015; Whiten et al. 1999). In addition, at *Bossou* this gesture is produced in play and frustration contexts (Sugiyama 1981), at *Mahale* in the sexual context (Nishida 1980), and at *Taï* only in combination with a drumming display (Boesch 1995; Luncz and Boesch 2015).

However, despite these research efforts, a detailed understanding of the impact of social transmission on gestural acquisition has been hampered by the lack of systematic comparative data across field sites and species. Although group-specific gestures have been reported in chimpanzees (Tomasello et al. 1997), gorillas (Pika et al. 2003; Tanner and Byrne 1999), and orangutans (Liebal et al. 2006) in captive groups, they represent an infrequent phenomenon in natural gestural interactions. In addition, a study investigating the onset of gestural production and early use in captive great ape infants showed that bonobo and chimpanzee infants share a considerably larger portion of their gestural repertoire with individuals of their respective age groups than with their mothers (Schneider et al. 2012). These results suggest that infants do not imitate the gestures of their mothers. Hence, they strengthen the findings of Hobaiter and Byrne (2011b) that ‘matrisyncratic’ gestural transfer does not play a role in great apes’ gestural acquisition (see Box 1). Moreover, a substantial portion of gesture types exchanged within mother–infant dyads is ‘one-way’ gestures. One-way gestures are produced by individual A to B but not by B to A (e.g. only the mother lowers her back to invite her infant to climb on her back but not vice versa; Goodall 1986; Halina et al. 2013), as would be predicted on any hypothesized ontogeny. The question remains whether recipients of one-way gestures can generalize this observational perspective and experience, and can become signalers themselves (e.g. lowering the back toward their own infants).

### The Ontogenetic Ritualization Hypothesis

The Ontogenetic Ritualization Hypothesis—also called conventionalization by others (e.g. Bates et al. 1979; Mead 1910; Vygotsky 1978)—originated as a response to the lack of imitation in great apes (Tomasello and Call 1997). It expands the ethological concept of phylogenetic ritualization to include the scenario of ontogenetic change. Inspired by Plooijs (1979, 1984) Social Negotiation Hypothesis, it proposes that an existing action transforms into a streamlined version—the communicative gesture—via repeated instances of interaction between two individuals.

A hypothetical scenario looks as follows:

1. Individual A uses a behaviour towards individual B (e.g. a chimpanzee infant, who wants to get carried, grabs the leg of her mother to climb up).
2. The recipient, individual B, reacts in a predictable way (e.g. the mother lowers her back to allow the infant to climb up).
3. On some subsequent occasion, individual B anticipates this action sequence on the basis of its first step (e.g. the mother lowering her back at the initial touch of her infant).
4. The initiator, individual A, learns over repetitions of this sequence to shorten its behaviour to just that initial step (e.g. touch as an intentional signal for eliciting the mother’s receptivity to carrying).

The Ontogenetic Ritualization Hypothesis predicts that any action could—in theory—be ritualized into a gesture, with different outcomes in different dyads for the same purpose. Thus, the lack of gestural uniformity among individuals, within and between groups, paired with the production of idiosyncratic gestures (see Box 1) is to be expected. The first studies of the ‘Leipzig Gesture group’ tested this hypothesis by focusing on levels of concordance in gestural repertoires of several captive groups of great apes (for an overview, see Call and Tomasello 2007). They showed that within-group variation consistently exceeded between-group variation, and dyads differed in relation to the degree of individual variation involved in gestural production. In addition, individuals produced idiosyncratic and one-way gestures. A recent study on gestural development in mother–infant dyads of captive bonobos tried to control for the impact of different contexts on gestural signalling by solely focusing on a single communicative function—to initiate leaving together to move to a new location with the infant clinging to the back or belly of the mother (Halina et al. 2013). The authors showed that dyads differed in the production of gesture types employed with relatively low degrees of concordance of gestural repertoires in the class of mothers or infants and very few gestures found in more than two or three individuals in a given class (Halina et al. 2013).

In contrast, other researchers argue that the Ontogenetic Ritualization Hypothesis is inadequate to explain gestural acquisition (Byrne et al. 2017; Perlman et al. 2012; Tanner et al. 2006) and/or has led to several misconceptions (Fröhlich et al. 2016b; Pika 2014). One issue concerns the assumption that gestures emerge from actions devoid of communicative meaning (Liebal and Call 2012). Several researchers failed to identify physically effective sequences of actions (Genty et al. 2009; Hobaiter and Byrne 2011b), which are supposed to become ritualized into a communicative signal (Tomasello et al. 1994). Since there are often several possible ways of achieving the same physical result, it is also unclear which features of the original action should be depicted by the streamlined gesture (Liebal and Call 2012). Moreover—although never explicitly stated by Tomasello and Call (1997)—some scholars assume that gestures acquired via Ontogenetic Ritualization cannot be generalized across dyads (Byrne et al. 2017; Halina et al. 2013). Such a view predicts an enormous amount of ‘work’ for each individual to acquire a gestural repertoire that is understood by the majority of its group members and conversely to also comprehend the meaning of conspecifics’ gestures directed towards itself (Byrne et al. 2017). In addition, the use of one-way gestures, idiosyncratic repertoires, and consequently no shared meaning within communities should be frequent phenomena (Pika 2014).

Furthermore, Byrne and colleagues (Byrne et al. 2017; Genty et al. 2009; Hobaiter and Byrne 2011b) stressed that the majority of studies and findings (e.g. presence of idiosyncratic and one-way gestures) supporting the Ontogenetic Ritualization Hypothesis are due to insufficient sampling efforts (shortage of observation hours and observation periods) rendering the assessment of individuals’ gestural repertoires virtually impossible.

## The Social Negotiation Hypothesis

### History of the Social Negotiation Hypothesis

Over forty years ago, Plooij (1978, 1984) formulated the Social Negotiation Hypothesis to explain the results of his observations on the developmental trajectory of communication in five mother–infant dyads at *Gombe*, Tanzania. This pioneering study was one of the first comparative studies that set out from the philosopher John Austin’s Speech Act Theory (Austin 1962) and its expansion by Bates et al. (1975). The theory centres around performative utterances and sees the issuing of a spoken utterance as the performing of a perlocutionary or illocutionary action. Perlocutionary speech acts are behaviours in which communication occurs only because the receiver is adept at interpreting the behaviour of the ‘sender’ (e.g. fear, excitement, curiosity). In other words, the signaller acts perlocutionary without the intention to communicate with the recipient. Contrarily, illocutionary speech acts, being immaterial, cannot result from the behaviour of any one individual impacting on another. Rather, illocutionary force assumes joint attention and meeting between two or more minds, who establish or negotiate a shared perspective on the world (Austin 1962). Bates et al. (1975) expanded this theory to communicative acts in general to embrace also gestural and bimodal communicative interactions. Here, illocutionary communicative acts involve the signaller’s understanding that a behaviour/signal can be used to manipulate interactants and may lead to a certain outcome (Bates et al. 1975). Plooij (1978, 1979, 1984) argued that—similarly to human infants—chimpanzees undergo a transition from perlocutionary to illocutionary acts at around 9–12 months of age. At this developmental stage, the chimpanzee infant is able to *maintain* an interaction, e.g. ‘play–tickling’. However, it is also able to *initiate* it, by using signals which have been conventionalized from previous social interactions (Plooij 1978). Plooij concluded that gestures in chimpanzees do not represent fully formed, innate signals but develop over the course of an individual’s interactional experience with its social environment. Gestures are thus acquired throughout ontogeny via a process of social negotiation (sensu Wittgenstein 1953).

### Mother–infant communication in chimpanzees living in natural environments

Developmental studies, using a longitudinal and cross-sectional (between- and within-subject) design, enable researchers to gain detailed insight into nonhuman primates’ communicative development, but are still rare outside of captive settings (Halina et al. 2013; Pika et al. 2003; Schneider et al. 2011; Tomasello et al. 1997). However, to shed light on gestural acquisition, it is mandatory to turn to populations living in their natural environments with active selection pressures at work (Boesch 2007). Recently, we (Fröhlich et al. 2016a, b, 2017) instigated the first systematic quantitative comparison of gestural communication and development across two geographically separated chimpanzee communities—*Kanyawara* community, Kibale National Park, Uganda, and *Taï South* community, Taï National Park, Côte d’Ivoire—and two different subspecies: *Pan troglodytes schweinfurthii* and *Pan troglodytes verus*. During the two study periods, the *Kanyawara* group comprised of 53 and 56 individuals and the *Taï South* community of 21 and 24 individuals. Both communities have been studied regularly since 1987 (Wrangham et al. 1992) and 1979 (Boesch and Boesch-Achermann 2000), respectively, with the chimpanzees being very well habituated to human observers. We were able to study the communicative behaviour of a total of 13 mother–infant dyads (seven from *Kanyawara* and six from *Taï South*) over two different field periods (*Kanyawara*: March–May 2013, March–June 2014; *Taï South*: October–December 2012, October–December 2013) per community and during two consecutive years. The age of the offspring ranged from nine to 69 months. At *Taï South,* one mother gave birth to another infant in the second field period, resulting in observations of twelve chimpanzee mothers and 13 infants. We applied a focal behaviour sampling approach (Altmann 1974) and particularly focused on communicative interactions in three distinct contexts—food sharing, mother–infant joint travel, and social play (for definitions of contexts and methodological details, see Fröhlich et al. 2016a, b).

We paid special attention to the main criticisms raised against methodological designs used in previous studies on gestural acquisition in captivity (e.g. shortage of observational periods, definition of idiosyncracy; Fröhlich et al. 2017; Genty et al. 2009; Hobaiter and Byrne 2011b). For instance, Hobaiter and Byrne (2011b) suggested that the assessment of the community repertoire of the *Sonso* community, Budongo, Uganda approached or had reached asymptote at approximately 15 h of active gesture time or approximately 150 days of field observation time (community size: *N* = 82). Although we applied a different observational data design and did not aim to assess the community repertoires, we ensured to maximize observation periods and field observation time. We observed all 13 dyads for a minimum of 150 days (in total 1155 h; *Kanyawara*: 214 h + 343 h; *Taï South*: 264 h + 334 h) of field observation time, resulting in a total of 169 h of video footage. Furthermore, Byrne and colleagues (Genty et al. 2009; Hobaiter and Byrne 2011b) had argued that differences in gestural repertoires of captive apes were simply premature assumptions, with repertoires yet to reach asymptote. For our repertoire^2^ analyses of mother–infant interactions, we therefore included only data of individuals, who had been observed for a minimum of 60 h, and whose gestural repertoires had reached an asymptote. For instance, the cumulative repertoires of gestures in the play context at *Kanyawara* and *Taï South* reached an asymptote after 14 and 20 days of field observation days, respectively (Fröhlich et al. 2016a).

Our first study, the ‘joint travel study’, focused on the single communicative function of joint travel initiation—to initiate leaving together to move to a new location with the infant clinging to the back or belly of the mother (sensu Halina et al. 2013). We particularly investigated whether gestural production is due to phylogenetic ritualization or learning (Fröhlich et al. 2016b). Statistical analyses are based on a total of 415 carry initiations (*Kanyawara*: *N* = 218; *Taï South*: *N* = 197; mean recordings per dyad: 33.2). The coding of the data set resulted in a total (number of cases) of 442 actions (*Kanyawara* mothers: *N* = 178, infants: *N* = 20; *Taï South* mothers: *N* = 204, infants: *N* = 40), 599 gestures (*Kanyawara* mothers: *N* = 337, infants: *N* = 22; *Taï South* mothers: *N* = 228, infants: *N* = 12), 51 bimodal (see Box 1; *Kanyawara* mothers: *N* = 2, infants: *N* = 28; *Taï South* mothers: *N* = 4, infants: *N* = 17), and 80 vocalisations (*Kanyawara* mothers: *N* = 3, infants: *N* = 39; *Taï South* mothers: *N* = 6, infants: *N* = 32; for further details, see Fröhlich et al. 2016b).

Since repertoire sizes of infants in this single context were too small for assessing concordance rates, we compared concordance rates in gestural repertoires of chimpanzee mothers within and between study groups. We applied the Dice coefficient (*D*_c_) (Dice 1945), which ranges from 0 to 1. While a value of 0 denotes that two given individuals share no gesture type, a value of 1 implies that the two compared gestural repertoires are identical. The results revealed considerable variability in gestural production, with only moderate (> 0.7) levels of concordance between the individual gestural repertoires of mothers of the same community. Levels of concordance were also moderate between mothers of different communities/subspecies, while group-specific gestures were absent. However, we observed three idiosyncratic gesture types, which were produced by different mothers across both study periods in this particular context. Additionally, we investigated whether the form and usage of gestures differed across development. In other words, does infant age influence signal production in both mothers *and* infants? We examined the effects of age and dyadic role on signal usage, while controlling for confounding variables like the mother’s parity, the infant’s sex, and the study site. We found that both mothers and infants were more likely to produce visual (see Box 1) gestures if infants were older. The production of tactile (see Box 1) gestures decreased with infant age. In addition, carry-initiating physical actions were produced more frequently by dyads with younger infants and decreased considerably with progressing development. These findings were consistent with our expectations: during development, the time spent in close maternal proximity decreases and infants become intentional agents, which are able to manipulate the attentional and possibly also the mental states of conspecifics (Pika and Mitani 2006; Plooij 1979; Tomasello et al. 2003). Indeed, as physical distance between mothers and their maturing infants and mobility increases, visual gestural communication, in addition to vocalizations, seems to become a crucial tool to support mother–infant coordination (Bard et al. 2005; Van Lawick-Goodall 1968b).

### The revised hypothesis of social negotiation

The findings of the joint travel study led us to conclude that meaningful and thus functional gestural signals do not represent simple innate, fully formed constant means of communication (sensu Byrne et al. 2017; Genty et al. 2009; Hobaiter and Byrne 2011b). Rather, their production is due to flexible online modifications, shaped over time, and thus the output of learning processes (Fröhlich et al. 2016b).

Given the observation of idiosyncratic gestures, the absence of group-specific gestures, and the considerable inter-individual variability, we argued that neither phylogenetic ritualization nor the prevailing learning hypotheses could convincingly explain the present results. We thus revisited the Social Negotiation Hypothesis (Plooij 1978; Wittgenstein 1953) and developed a revised version. The revised hypothesis postulates that gestures emerge from an exchange of social behaviours between interactants, resulting in mutual understanding that specific behavioural patterns can be used as communicative tools to transfer distinct information and to achieve desired goals. Therefore, the creation of gestural signals does not necessarily begin with shortening of an action sequence (sensu Tomasello and Call 1997) but rather via fully formed behaviours through repeated exchanges with social partners. Here, interactants also learn that distinct social constellations and contexts assign different meaning/s to gestural signals and can lead to different outcomes. In contrast to the Ontogenetic Ritualization Hypothesis, this knowledge can be directly used in interactions with unfamiliar partners. It thus facilitates the most efficient and least costly communication transfer while in parallel being open to online adaptation and refinement. Similarly, Bard et al. (2014) recently proposed that most gestures emerge from meaningful social interactions through inter-subjective processes. They vary according to the social context (Fogel and Thelen 1987) and may be subject to continuous communicative validation (Bard et al. 2014).

In contrast to the predictions of the Phylogenetic Ritualization Hypothesis (Byrne et al. 2017), the potential repertoire of available gesture types is only limited via anatomical features and movement constraints of a given species (Pika 2014), the respective communicative scenario (e.g. short- versus long-distance communication, interaction partner), social context, and recipient affordances (attentional state, location, posture, and distance to recipient; Pika 2014; Wittgenstein 1953). While the subset of regularly employed gesture types may indeed be fine-tuned during development (Hobaiter and Byrne 2011a; Pika et al. 2003; Tomasello et al. 1994), interactants mutually shape—or in our words ‘socially negotiate’—the outcome of each gestural interaction in real time. Hence, the emerging gestural output represents a manifold variation in size, scope, strength, location, and orientation of a given gesture. For instance, although researchers apply the single umbrella term touch to refer to light and brief (under 2 s) contact of the palm and/or fingers of signallers on the body of the recipient, each gestural performance of a touch gesture, by a given signaller, is a highly variable online adjustment (Perlman et al. 2012). Similarly, loud and exaggerated scratching gestures—directed scratches—used in the chimpanzee community *Ngogo* to negotiate role reversal and cooperation during grooming (Pika 2014; Pika and Mitani 2006) are characterized by too many variations in size, scope, location, and orientation of gestures to qualify as constant in form over time. Furthermore, since they seem to fulfil different functions and convey different meanings in other wild chimpanzee communities (e.g. they have been reported at *Gombe* to be produced as one-way gestures by mothers towards their infants to solicit leaving a location together; Goodall 1986), they strengthen our argumentation that social negotiation plays a crucial role in enabling and transmitting communicative meaning—and thereby possibly communicative culture—within great ape communities (see also van Leeuwen et al. 2012).

### Effects of the social environment on communicative development

Initially, the revised Social Negotiation Hypothesis was proposed as a consequence of our findings on a single communicative function and dyad: joint travel solicitations between mother–infant dyads (Fröhlich et al. 2016b). The joint travel study, however, did not account for the influence of familiarity, interaction partner, social exposure, and thus interactional experience, which are also crucial indicators of whether or not learning plays a role in gestural acquisition. Hence, the next step in our research was to investigate interactions outside the mother–infant bond and to test the way infants apply gestures to influence the behaviour of non-maternal conspecifics, such as siblings, peers, and unrelated adults. Most importantly, our findings provided strong support for the hypothesis that learning and interactional experience with social partners play a crucial role in gesture acquisition. Here, we summarize the central objectives and findings of two recent studies (the play study and the context study). In these two studies, we applied the same methodological design as used in our joint travel study and also collected the data in the same two communities of chimpanzees in the wild (for details, see Fröhlich et al. 2016b).

In the play study, we investigated communicative exchanges during play interactions and examined the influence of demographic factors, namely the interactants’ age, sex, and kin relationship on gestural signalling (Fröhlich et al. 2016a). Analyses are based on a total of 618 play interactions (Kanyawara: *N* = 352; *Taï South*: *N* = 266). The coding of the videos resulted in a total (number of cases) of 1174 gestures (*Kanyawara*: *N* = 761; *Taï South*: *N* = 413), including 109 audible (*Kanyawara*: *N* = 74; *Taï South*: *N* = 35), 646 tactile (*Kanyawara*: *N* = 417; *Taï South*: *N* = 229), and 419 visual gestures (*Kanyawara*: *N* = 270; *Taï South*: *N* = 149; for further details, see Fröhlich et al. 2016a). Among these were 229 cases of object-associated gestures (see Box 1; *Kanyawara*: *N* = 125; *Taï South*: *N* = 104) and 74 cases of self-handicapping gestures (see Box 1; *Kanyawara*: *N* = 27; *Taï South*: *N* = 47).

**Fig. 1:**
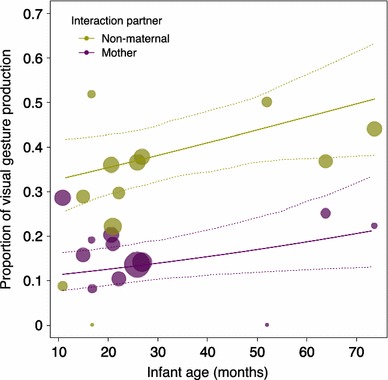
Influence of mean age and interaction partner on the employment of visual gestures in chimpanzee infants. Depicted are raw proportions, separately for each infant against its mean age. The area of the dots corresponds to the sample size per individual and interactional dyad (range 5–319). The solid and dashed lines represent the fitted model and confidence interval based on all other covariates and factors centred to a mean of zero

We analysed the form of signals in relation to developmental phase, context, and interactional partner and found a strong effect of age on the production of different gesture categories: visual and audible gestures were produced at the expense of tactile signals with increasing age. In addition, there was a sex difference in gestural usage, with males employing more tactile signals than females. Tactile gestures differ from visual gestures in both physical effectiveness and potential demonstration of physical strength, suggesting a sex difference in signal directness in terms of the level of physical contact involved. In line with these results, a recent study by Lonsdorf et al. (2014) reported pronounced sex differences in the social play interactions of chimpanzees of the *Gombe* community, Tanzania. Male individuals showed higher playing rates at earlier ages, a more diverse network of social partners, and a higher frequency of interaction with adult males. The authors argued that social experience is more important for young chimpanzee males, since the formation of social bonds and apprenticeship during development influences dominance status in adulthood (Matsuzawa et al. 2001; Mitani 2009; Muller and Mitani 2005). Kin relationships (categorized as maternal, maternal kin, and non-kin relationships) also had a profound impact on gesture performance, with tactile gestures being more and visual gestures less frequently employed in interactions with mothers than with other individuals (i.e. both maternal kin and other partners; see Fig. 1). Even more importantly, immature chimpanzees flexibly adjusted their gestural output according to key attributes of gesture recipients, with age difference and relationship between interactants having a strong impact upon signal production. For instance, object-associated (e.g. branch-shaking) and self-handicapping (e.g. poke while lying in a supine position) gestures were of crucial importance for initiating play with partners of the same age and younger, respectively. The play study demonstrated that play interactions with peers and other non-maternal individuals may serve as an essential training ground to provide an opportunity for experiencing the production and usage of communicative signals. Here, individuals can test the effectiveness of, and practice, gestural signalling that might be of vital importance for negotiating and maintaining social relationships in later life.

In the context study, we compared gestural signalling across three different communicative contexts—food sharing, joint travel, and social play (Fröhlich et al. 2017). Statistical analyses are based on 1120 high-quality recordings of communicative interactions collected in the three behavioural contexts (food sharing: *N* = 260; joint travel: *N* = 392; social play: *N* = 468). We identified a total of 1066 gestures, with 301 infant gesture cases produced during the context of food sharing, 77 to initiate joint travel, and 688 gesture cases produced in the play context. Overall, we described a total of 55 different gesture types (*Kanyawara*: *N* = 49; *Taï South*: *N* = 47), with seven types being exclusively produced in the food sharing context and 37 in the play context. Five gesture types were utilized in all three contexts, four in both the food sharing and the play contexts. Two gesture types were utilized in both the joint travel context and the play context.

**Fig. 2:**
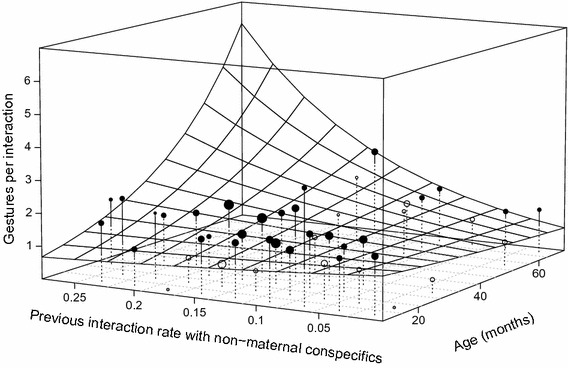
Influence of age and interactions with non-maternal conspecifics on the gesture frequency of infants. The surface represents results from the GLMM with all covariate centred to a mean of zero; the points depict the average repertoire size per cell of the surface. Values above the fitted model are depicted as filled points, values below as open points. The volume of the points corresponds to number of samples per cell. Graph derived from Fröhlich et al. (2017)

In the context study, we especially examined the role of social exposure, namely behavioural context, interaction rates, and maternal proximity, on infant gestural production (Fröhlich et al. 2017). Specifically, we investigated the sources of variation in the frequency at which infants produced gestures, gesture sequences, and infants’ repertoire sizes. To quantify the influence of previous social interactions with mothers and other conspecifics on the production of gestures, we used a novel combination of video recordings and focal scans (Altmann 1974; for details of study methods see Fröhlich et al. 2017). Overall, we found that social play was the context in which the majority of gestural signals and gesture types were employed (see for similar findings; Liebal et al. 2006, 2013; Pika et al. 2003, 2005; Tomasello et al. 1997). Of particular relevance for the present paper is the finding that previously experienced interactions with conspecifics—but not mothers—had a positive influence on gestural frequency and repertoire size. This result was further supported by the fact that gestural repertoire size increased with the number of interaction partners encountered in the previous month of life (see Fig. 2). These findings suggest that the development of intentional gesturing in chimpanzee infants depends highly on the complexity of the surrounding social world and its opportunities to interact with conspecifics (Fröhlich et al. 2016a; Plooij 1978).

In sum, the play and context studies strengthen the argument that communicative development in chimpanzees heavily relies on the infants’ social environment via interactions with maternal kin and other non-related social partners as soon as infants become more independent (Van Lawick-Goodall 1968b). Since communication usually takes place within complex social interactions, it is to be expected that individuals rely on input from their social environment before communicative skills fully manifest themselves (Liebal et al. 2013). Our results provide the first evidence that the documented early sex difference in sociability in chimpanzees is also apparent in the development of communicative signalling. These differences between males and females possibly reflect the differential importance of early socialization (Murray et al. 2014). Our findings further corroborate the crucial role of interactional experience with different social partners for communicative development. This empirical link between social–interactional experience and gestural performance suggests that learning via repeated and diverse social interactions comprises the primary mechanism by which young apes acquire their gestural repertoires (see also Bard et al. 2014). While the mother–infant relationship is critical for normal social development (Maestripieri et al. 2009), research also demonstrates that early socialization in the wider social environment is essential for developing social competency later in life (Hamilton 2010; Parker and Asher 1987). In the fission–fusion social structure characteristic of chimpanzees (Nishida 1970), the mother can actively influence her offspring’s social experience through selective subgrouping (Murray et al. 2014). Our results thus strongly suggest that infants of social mothers have a head start into the complexity of social life by experiencing higher numbers of social contacts and experiences. From a very early age, chimpanzees seem to exploit these social opportunities with the number of social partners increasing with offspring age and distance to mothers (Lonsdorf et al. 2014; Murray et al. 2014).

## Outlook

### Methodological aspects

Recent comparative work in captive and natural environments provides evidence that the developmental approach is crucial to gain an in-depth understanding of communicative abilities and cognitive underpinnings of our closest living relatives (Bard and Leavens 2014). In a similar vein, we thus hope to inspire future research testing the predictions and implications of the Social Negotiation Hypothesis in more detail. Research efforts need to focus particularly on three methodological aspects to pinpoint the major routes of gesture acquisition.

First, a longitudinal and cross-sectional approach will allow an assessment of how gestural production and usage unfolds over time in individuals, paired with a within- and between-site comparison of study individuals. For this study design, it is crucial to use the method of within-subject centring (van de Pol and Wright 2009) to avoid the effect of pseudoreplication, and to disentangle whether the effect of infant age is particularly relevant within and/or between infants. Pseudoreplication refers to the use of inferential statistics to test for treatment effects for data from experimental or observational studies where either treatments are not replicated or replicates are not statistically independent (e.g. repeated observations of the same individual). Within-subject centring means that the effect of infant age is included in the form of two variables in statistical models: (1) the average age of each individual infant (‘between age’), and (2) the difference between the individual infant’s actual age and its average age (mean centred or ‘within age’).

Second, the independent collection of data on behavioral and communicative interaction rates and the number of interaction partners will enable researchers to investigate in detail the influence of social and communicative exposure and opportunities on gestural production. For instance, this can be measured via frequency, sequential use, meaning, and context-specific and general repertoires of gestures. In previous studies, we used focal scans conducted in 15-min intervals, but all-occurrence sampling of interactions might be an even more accurate sampling method. Focal scan data complementing video recordings allow for tracing both the social (e.g. interaction rates and partners) and spatial independence (e.g. maternal proximity) of immature apes, which optimally complement the fine-grained analysis of gestural production via video recordings. Therefore, by using this method multiple domains of development can be assessed simultaneously.

Third, previous studies differ to a lesser or a greater degree in the level of ‘splitting’ and ‘lumping’ of behavioural categories to assign and distinguish between different gesture types (Call and Tomasello 2007; Genty et al. 2009; Goodall 1986; Graham et al. 2017; Hobaiter and Byrne 2011b; Nishida et al. 1999; Roberts et al. 2014). In addition, some descriptions of widely used gesture types refer to a stringent, distinctive bodily movement—such as the terms chest beat or slap ground—while other gesture types incorporate highly variable movements under the same umbrella term (e.g. touch). Originally intended to cluster the behaviour of animal species into recognizable and reliable units (Kummer 1968; van Hooff 1967, 1973; Van Lawick-Goodall 1968a, b), this approach may have resulted in overlooking fine-grained gestural differences. We thus may also have missed evidence verifying the role of learning and ‘culture’ in gestural signalling. Furthermore, the creation of different gesture types should always be closely accompanied by an assessment of whether this difference exists in the eyes of the human beholder only or whether signals carry different information for signallers and/or recipients.

### Studying developmental trajectories across modalities

Since vocal production of great apes and other nonhuman primates is thought to be highly constrained and non-intentional (but see Crockford et al. 2012; Schel et al. 2013 for short-distance vocalizations in chimpanzees), the most predominant hypotheses on signal acquisition have focused solely on gestural signalling. Given that the impact of social experience on vocal development has been widely overlooked (but see Katsu et al. 2017; Laporte and Zuberbühler 2011; Snowdon 2017; Snowdon and Hausberger 1997), it is not only vital to understand the role of learning for vocal but also bimodal signal production (see also Higham and Hebets 2013) in non-human primates. Our current knowledge on the development and cognitive underpinnings of communicative skills in great apes is thus highly restricted to unimodal signal production (Slocombe et al. 2011; see for recent work on bimodal usage Fröhlich et al. 2016b; Hobaiter et al. 2017; Hopkins et al. 2007; Luef and Pika 2017; Pollick et al. 2008). Very little is known, however, about the frequency of bimodal signal production (Fröhlich et al. 2016b; Hobaiter et al. 2017), the usage (Luef and Pika 2017), and the function and meaning of bimodal versus unimodal signalling. In addition, we also do not know whether and how the developmental trajectories of bimodal and unimodal signals may differ. The few existing studies suggest that different cognitive mechanisms may be involved (Katsu et al. 2017; Laporte and Zuberbühler 2011; Snowdon and Hausberger 1997). The social cognitive skills, which enable the linkage between different signals and signal categories, seem to develop later in ontogeny than those used in unimodal signal production. This pattern has so far only been confirmed in human children, with gestural usage preceding bimodal combinations embodying combinations of gestures and spoken words (Bates et al. 1975; Iverson and Goldin-Meadow 2005). However, relatively little is known about the onset of combinations of intentional vocalizations (Oller et al. 2013) and gestures in human children. However, recent observational and experimental work showed that bimodal combinations of pointing gestures and point-accompanying vocalizations occur already at the ages of eleven (Leroy et al. 2009) and 14 months, respectively (Grünloh and Liszkowski 2015). Gestures therefore precede the use of spoken words, but may be accompanied from their early onset on by intentional vocalizations. Similarly, Liebal et al. (2013) suggested that bimodal signalling could appear first and may be later fine-tuned and or replaced by the most effective unimodal signals. We recently provided some evidence for the latter explanation in chimpanzees by showing that—at least in the specific communicative function of joint travel—a developmental shift from predominantly vocal to gestural signalling takes place (Fröhlich et al. 2016b). The vocal-gesture shift may be selected for in evolutionary urgent contexts (e.g. hunting and patrol in chimpanzees, Mitani 2009), where predators or members of neighbouring groups severely impact upon individuals’ survival and reproductive success.

## Conclusions

With this article, we make the case that the most predominant perspectives of gestural acquisition—Phylogenetic Ritualization, Social Transmission via Imitation, and Ontogenetic Ritualization—do not satisfactorily explain the variability and distribution observed in chimpanzees’ (and probably also other great apes’) natural communicative gesturing. In contrast, our results suggest that the role of interactional experience and social exposure on gestural acquisition and communicative development of chimpanzees has been severely underestimated. We propose that the production and usage of communicative gestures highly depend on social negotiation between interactants and are open to online adaptation and refinement. This results in a shared understanding that specific behaviours can be used to achieve communicative goals and carry distinct meanings linked to particular social contexts. Chimpanzees' gesture acquisition thus highly matches the learning process suggested by Acredolo and Goodwyn (1988; Goodwyn and Acredolo 1993; see also Caselli 1990) for human children: gestural production and usage are learned within social routines with familiar interaction partners. Every comparative researcher interested in developmental questions wishes to have access to any possible behaviour the studied individuals have ever produced in their lifetime. This aim—besides the tremendous recent technical revolution—can, however, not yet be met in species living in their natural environments. Thus, although our results are based on a relatively small sample size and a systematic, quantitative comparison of gestural signalling across two wild chimpanzee communities only, we sincerely hope that this so far unprecedented approach furthers more comparative research. Future projects should aim (1) to carry out quantitative comparisons across several communities, species, and taxa, as well as (2) longitudinal studies of signal development allowing fine-grained analyses of signal production and usage. We especially need to move away from a clustering approach where distinct movements are loosely assigned into specific categories (e.g. touch, reach), resulting in overlooking individual differences, function, online adjustment, and downplaying the impact of social learning on gestural acquisition.

By applying a comparative, developmental approach to understand the role of learning in communicative signalling of our closest living relatives, we hope to have also stimulated future research shedding additional light on learning mechanisms involved in early gesturing of human children (Acredolo and Goodwyn 1988; Child et al. 2014; Tomasello 1996). In addition, the ability to attribute new meaning to signals and to dissociate existing signals from behavioural domains, ends, and contexts—called semantization (Wickler 1967)—may have been selected for in those species only which heavily rely on cooperation and the negotiation of social relationships (Pika 2016; Pika and Bugnyar 2011).

## Footnotes

1 From here on gestures and vocalizations are depicted in small capitals.

2 Concerning our three studies mentioned here, the term ‘repertoire’ is used to refer to those gestures observed during the observation period in interactions between mother–infants and/or infant-peer interactions.
